# Elucidating the Role of the Complement Control Protein in Monkeypox Pathogenicity

**DOI:** 10.1371/journal.pone.0035086

**Published:** 2012-04-09

**Authors:** Paul N. Hudson, Joshua Self, Sonja Weiss, Zachary Braden, Yuhong Xiao, Natasha M. Girgis, Ginny Emerson, Christine Hughes, Scott A. Sammons, Stuart N. Isaacs, Inger K. Damon, Victoria A. Olson

**Affiliations:** 1 Poxvirus and Rabies Branch, Division of High Consequence Pathogens and Pathology (DHCPP), National Center for Emerging and Zoonotic Infectious Diseases (NCEZID) and Biotechnology Core Facility Branch, Division of Safety Research (DSR), National Center for Emerging and Zoonotic Infectious Diseases (NCEZID), Centers for Disease Control and Prevention, Atlanta, Georgia, United States of America; 2 Division of Infectious Diseases, Department of Medicine, University of Pennsylvania School of Medicine, Philadelphia, Pennsylvania, United States of America; Lisbon University, Portugal

## Abstract

Monkeypox virus (MPXV) causes a smallpox-like disease in humans. Clinical and epidemiological studies provide evidence of pathogenicity differences between two geographically distinct monkeypox virus clades: the West African and Congo Basin. Genomic analysis of strains from both clades identified a ∼10 kbp deletion in the less virulent West African isolates sequenced to date. One absent open reading frame encodes the monkeypox virus homologue of the complement control protein (CCP). This modulatory protein prevents the initiation of both the classical and alternative pathways of complement activation. In monkeypox virus, CCP, also known as MOPICE, is a ∼24 kDa secretory protein with sequence homology to this superfamily of proteins. Here we investigate CCP expression and its role in monkeypox virulence and pathogenesis. CCP was incorporated into the West African strain and removed from the Congo Basin strain by homologous recombination. CCP expression phenotypes were confirmed for both wild type and recombinant monkeypox viruses and CCP activity was confirmed using a C4b binding assay. To characterize the disease, prairie dogs were intranasally infected and disease progression was monitored for 30 days. Removal of CCP from the Congo Basin strain reduced monkeypox disease morbidity and mortality, but did not significantly decrease viral load. The inclusion of CCP in the West African strain produced changes in disease manifestation, but had no apparent effect on disease-associated mortality. This study identifies CCP as an important immuno-modulatory protein in monkeypox pathogenesis but not solely responsible for the increased virulence seen within the Congo Basin clade of monkeypox virus.

## Introduction

Several members of the Orthopoxvirus genus such as cowpox, monkeypox and vaccinia viruses continue to be human pathogens of significant worldwide concern. Variola virus, an obligate human pathogen, was eradicated through a World Health Organization (WHO) global immunization campaign. Prior to 1970, monkeypox virus was characterized as a non-human primate pathogen, until human monkeypox cases were reported in a number of West African countries and in the Congo Basin. Over the next several years, WHO surveillance activities characterized a large number of human monkeypox cases [1] in the Congo Basin country, now known as the Democratic Republic of Congo (DRC), which continues to have reportable monkeypox disease today [2]. The clinical manifestations of human monkeypox were found to be similar to those of discrete, ordinary smallpox [3]. More recently, clinical and epidemiological evidence suggested that there were virulence and interhuman transmissibility differences between human monkeypox caused by West African or Congo Basin origin viruses [4]. Furthermore, whole genome sequencing confirmed that monkeypox viruses formed two distinct genetic groupings, which correlated with their geographic origins, the West African and Congo Basin clades [4], [5]. In 2003, the first report of human monkeypox disease outside of Africa occurred within the U.S., highlighting the potential for monkeypox virus spread into a number of non-endemic regions, and the importance of gaining a more detailed understanding of monkeypox [6]. In the 2003 U.S. outbreak, after controlling for age and vaccination status, disease caused by a strain of West African clade monkeypox virus was characterized as less virulent and less interhuman transmissible than that previously ascribed to human disease caused by Congo Basin clade viruses [4]. In a small study of non-human primates, a similar difference in the virulence of strains from each of the two monkeypox virus clades was described [5].

The U.S. reported cases all stemmed from the importation and distribution of exotic pets. In these cases, human owners contracted the disease from their infected North American black-tailed prairie dog pets (*Cynomys ludovicianus*), which had been previously co-housed with imported monkeypox virus-positive African rodents [6], [7]. Subsequent research characterized monkeypox virus challenge of prairie dogs as a useful animal model for systemic orthopoxvirus infection, causing a rash illness with a disease progression similar to systemic human disease [8]. This model also recapitulates aspects of the differences in virulence between the two monkeypox virus clade viruses.

Genomic comparisons of strains from the Congo Basin and West African clades revealed multiple deletions and fragmentations of open reading frames in the West African strains of monkeypox virus that may contribute to its reduced virulence. Of note, the more virulent Congo Basin clade strains of monkeypox virus encode a homologue of the vaccinia virus complement control protein (CCP), annotated as C3L, while the West African clade strains do not [4], [5]. Although the monkeypox virus CCP homolog is truncated compared to the vaccinia virus CCP, studies have shown it retains certain complement inhibitor function [9].

The complement system is a non-adaptive network of cell-associated and secreted effector proteins that are important to the initiation of an inflammatory response. The two primary routes by which complement is activated are the classical and alternative pathways. Both of these pathways lead to the formation of the C3 convertase complex, which is composed of either C4b and/or C3b proteins. These C3 convertases proteolytically cleave the ever-present C3, resulting in further deposition of C3b, assembly of more C3 convertases and formation of the C5 convertase. This convertase cleaves C5 to C5b, which is necessary for formation of the membrane attack complex. The activation of complement leads to coating of virus particles with complement for the potential lysis of enveloped viral particles and virus-infected cells. The complement system is also important in virus neutralization by coating the exterior of viral particles with C3b, marking it for phagocytosis by macrophages and dendritic cells, and thus stimulating the presentation of antigens to the adaptive immune system. It has also been documented that this C3b coating prevents efficient attachment and entry of the viral particle into host cells [10]. Several cleavage products of the complement cascade, such as C3a and C5a, are chemotactic mediators involved in the recruitment of pro-inflammatory granulocytes and lymphocytes to the site of infection [11], [12], [13].

CCP homologues have been identified in vaccinia virus, known as VCP (vaccinia virus complement control protein); variola virus, known as SPICE (smallpox inhibitor of complement enzymes); ectromelia virus; and cowpox virus where it is referred to as IMP (inflammation modulatory protein) [13], [14], [15], [16], [17], [18]. These homologues are capable of inhibiting complement through protein binding and serving as a co-factor in cleavage of the C3b and C4b moieties and accelerating the decay of convertases in both the classical and alternative pathways [9], [14], [15], [19], [20], [21], [22], [23], [24]. The complement control protein homologue in vaccinia virus, VCP, is the prototypical poxvirus mimic of the mammalian regulators of complement (RCA) class of proteins. Like members of the RCA family, VCP is composed of four repeating short consensus repeats (SCR) with 30–40% identity to human RCAs [13], [14]. It is a major secreted protein in vaccinia-infected cells and inhibits early steps in complement activation [13], [14], [19], [20]. Due to the presence of SCR4, VCP also exhibits C3-convertase decay accelerating activity, resulting in a more rapid breakdown of these protein complexes, which are an integral part of the complement cascade [9], [12]. In several experiments, it was found that VCP was able to protect vaccinia virus against host complement-mediated neutralization [20], [25]. Vaccinia mutants lacking VCP displayed a measurable attenuation in its pathogenicity when intradermally injected in mice, rabbits and guinea pigs [20], [26], [27]. The CCP homologue in monkeypox virus is truncated early in the fourth SCR due to a single nucleotide deletion that results in chain termination [5]. Lacking the complete 4th SCR region, the monkeypox homologue lacks the C3 convertase decay accelerating activity found in VCP but retains C3b and C4b cofactor functions [9].

The inhibition of host complement pathways by viral CCPs is believed to abrogate the recruitment of pro-inflammatory cells to the site of initial infection, and slow the development of a robust host response to the pathogen [28], [29], [30]. The ability of a virus to secrete a protein which impairs the early innate immune response of the host to the developing infection would improve the ability of the virus to maintain viability in the host. The inclusion of CCP in the more virulent Congo Basin clade of monkeypox, and the lack of this gene product in the West African clade, thus makes it a protein which hypothetically may determine virulence differences between the two clades.

To understand the role of CCP in monkeypox disease pathogenesis, we constructed recombinant viruses, one incorporating the CCP gene under its native promoter in the West African/USA monkeypox virus and one removing the gene from the Congo Basin monkeypox virus strain. Western blot analysis confirmed the absence of CCP in the Congo Basin recombinant and the presence of CCP in the West African recombinant. We evaluated the virulence of the Congo Basin deletion mutant in comparison to its parental strain and to the recombinant West African clade expressing CCP to its parental strain using the black-tailed prairie dog challenge model [8]. The data presented here will provide a greater understanding of the pathogenesis of monkeypox virus, the importance of CCP for monkeypox virulence, and identify targets for effective vaccine design strategies and anti-viral treatments.

## Results

### Construction of ROCΔCCP and USA+CCP

**Figure 1:**
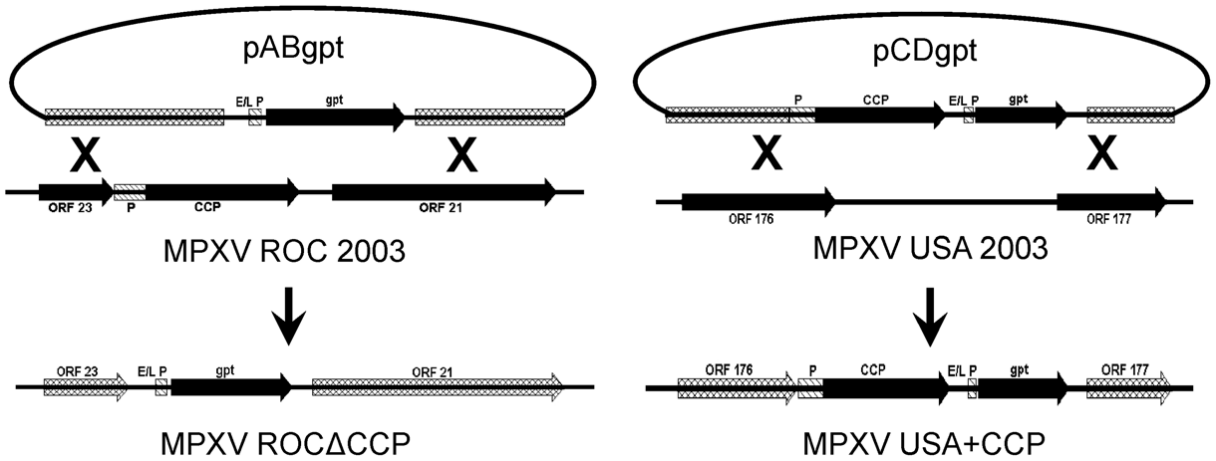
Constructs used for recombinant virus formation. Plasmid maps of pABgpt and pCDgpt illustrate the recombination sites within ROC 2003 and USA 2003 genomes. For both recombinant strains, gpt expression was controlled by a synthetic early-late promoter sequence. In the recombinant USA+CCP, the *ccp* gene was under its own native promoter from ROC 2003, and the insert was located in the non-coding region between open reading frames 177 and 176 of USA 2003.

Two plasmid constructs were made, one to incorporate CCP in the West African USA 2003 strain, and one to remove the gene from the Congo Basin ROC 2003 strain. The insertion of the CCP gene into USA 2003 clade was only begun after careful consideration and discussions with the institutional biosafety committee, the institutional animal care and use committee, and the select agent program. Approval was obtained since insertion of the gene encoding the CCP into the strain of virus that lacked its expression was an appropriate control to determine its function in virulence. Development of the ROCΔCCP and USA+CCP recombinant viruses required the incorporation of the selectable *gpt* gene marker from the pelP1-gpt vector. The *gpt* expression cassette was placed under the control of a vaccinia-derived synthetic early-late promoter (Figure 1). The complement control protein in ROC 2003 was knocked out by double homologous recombination at two flanking loci, deleting the gene and simultaneously conferring mycophenolic acid resistance to the recombinant. The USA+CCP recombinant was created by incorporating the Congo Basin strain CCP gene under the control of its predicted upstream promoter into the intergenic region between ORFs 176 & 177 (Figure 1).

Recombinants were analyzed for second site mutations by whole genome sequencing. Comparison of the USA+CCP genome (10,890 reads) to the published sequence showed one single nucleotide polymorphism (SNP) and one single nucleotide deletion, with neither located in coding or promoter regions. Mapping the sequencing reads from ROCΔCCP to the published ROC 2003 genome resulted in two contiguous sequences (contigs) (11,779 reads) separated by the deleted CCP region. Nucleotide comparisons revealed two SNPs. These SNPs affected a gene fragment of the A-type inclusion (ATI) protein causing an Asp to Val change at amino acid position 388 and a Val to Asp at position 386. These SNPs occurred in a repeat rich region of the truncated protein. It has been previously established that monkeypox viruses do not produce A-type inclusion bodies due to a frameshift mutation that causes premature truncation [4], [31]. Studies where the *ati* gene in cowpox virus was knocked out and compared to wild-type in mice have demonstrated limited effects on pathogenesis, adding to its classification as a non-essential protein product [32]. Since these two SNPs are within a truncated, non-essential gene, the alterations should not have a significant effect on the systemic disease pathogenesis of this recombinant.

### Phenotype confirmation of the recombinants and *in vitro* expression and biologic characterization

**Figure 2:**
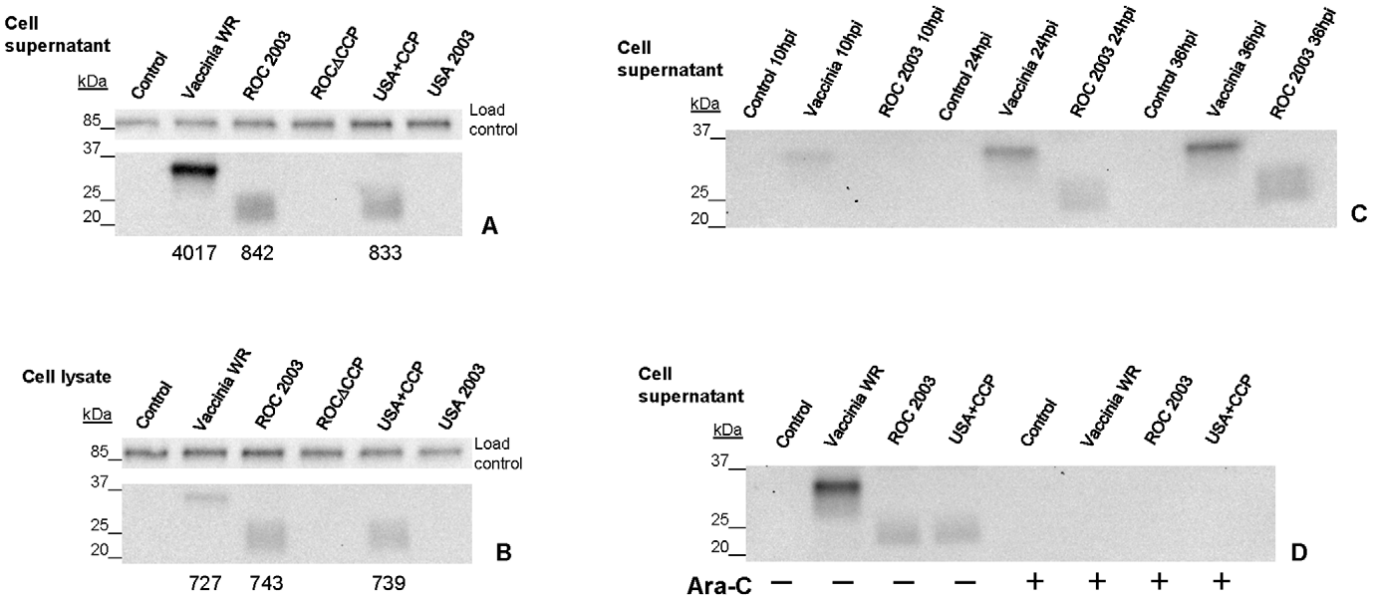
*In vitro* expression of CCP. BSC-40 cells were infected with the parental monkeypox virus (ROC 2003 or USA 2003), the recombinant monkeypox virus (ROCΔCCP or USA+CCP), or vaccinia WR. At 24 hpi, supernatants (A) and cell lysates (B) were harvested and probed with polyclonal α-VCP antibody. Complement control protein homologues in monkeypox virus (∼24 kDa) and vaccinia WR (∼35 kDa) were detected. Human transferrin (85 kDa) was used as the load control for each well. Numerical values below each lane in blots A and B represent the intensity value of the band using the Image Lab 3.0 band density tool. Each lane was loaded with (3 µg/ml) concentrated supernatant or cell lysate. BSC-40 cells were infected with either the ROC 2003 strain of monkeypox virus or vaccinia WR and supernatant was harvested and replaced with fresh media at the indicated times (C). The harvested supernatants were analyzed using a polyclonal α-VCP antibody. VCP expression was first detected in cell supernatants at 10 hpi and monkeypox CCP expression at 24 hpi. BSC-40 cells were infected with the parental monkeypox virus (ROC 2003 or USA 2003), the recombinant monkeypox virus (ROCΔCCP or USA+CCP), or vaccinia WR in the presence (+) or absence (−) of Ara-C. At 24 hpi, supernatants (D) were harvested and probed with polyclonal α-VCP antibody. Expression of CCP in the presence/absence of Ara-C in the West African recombinant monkeypox strain was similar to the Congo Basin strain.

Complement control protein expression in the USA+CCP recombinant and its absence in the ROCΔCCP knockout recombinant were evaluated by Western blot. Cell extracts and supernatants were prepared from infected BSC-40 cells at 24 hours post infection (hpi). The cell extracts and supernatants (concentrated ∼5X) were probed with rabbit-derived polyclonal antisera targeted against the vaccinia complement control protein (Figure 2A, 2B). Vaccinia-infected cell supernatants and cell extract samples were treated similarly to provide a positive control. In Western blot analysis, the expected size difference between the 35 kDa vaccinia CCP homologue and 24 kDa CCP in ROC 2003 and USA+CCP strains was readily apparent. CCP was not evident in the concentrated supernatants of ROCΔCCP or USA 2003-infected cells and the presence of CCP was equivalent in the cell extracts or concentrated supernatants from USA+CCP and ROC 2003-infected cells (Figure 2A and 2B). Quantification of the secreted monkeypox CCP (USA+CCP or ROC 2003), based on Western blot, was approximately five times lower than that detected from vaccinia WR infected cell supernatants (Figure 2A). There was similar accumulation of CCP within the ROC 2003 and USA+CCP cell extracts versus the vaccinia cell extract (Figure 2B). Importantly, there were no significant differences in CCP levels (supernatant or cell extract) between the ROC 2003 and USA+CCP recombinant at 24 hours post infection.

To determine at what stage of infection CCP was expressed, we used Ara-C, which results in the inhibition of late protein expression [33]. Cells were infected with either the ROC 2003 strain of monkeypox virus or vaccinia WR in the presence or absence of Ara-C, and supernatant was harvested and replaced with fresh media at the indicated times (10, 24, and 36 hpi, Figure 2C). The collected supernatants (∼5X concentrated) were analyzed by Western blot using a polyclonal anti-VCP antibody. Samples taken at earlier times (2, 4, and 6 hpi) were negative for CCP and VCP expression in the absence of Ara-C (data not shown). Growth of the virus from 6 to 10 hpi, allowed sufficient time for VCP to accumulate and be detected by Western blot, meanwhile monkeypox CCP expression was absent. In the sample collected at 24 hpi, the monkeypox homologue was first detected (Figure 2C). The addition of Ara-C eliminated the ability to detect VCP and monkeypox CCP by Western blot at any time point (data not shown). To evaluate the expression of monkeypox CCP in the USA+CCP recombinant, we chose a single time point (24 hpi) that based upon our time course experiment would have a sufficient amount of CCP expression. Western blots of supernatants (∼5X concentrated) from BSC-40 cells infected with USA+CCP MPXV, ROC 2003 MPXV, or vaccinia WR in the presence/absence of Ara-C were probed with anti-VCP rabbit polyclonal sera (Figure 2D). We found that USA+CCP and ROC 2003 accumulated similar levels of secreted CCP at 24 hours (Figure 2D). The CCP gene in USA+CCP is expressed at late times and likely under the control of the predicted upstream late promoter, derived from the ROC 2003 strain of monkeypox virus, contained within the construct.

**Figure 3:**
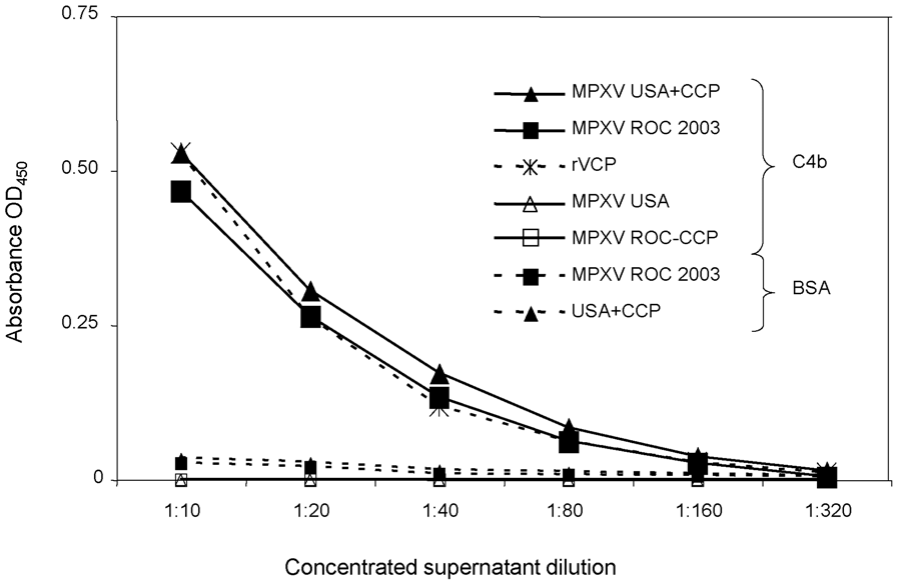
C4b binding activity of monkeypox virus CCP. Assay measuring the binding of MPXV complement control proteins from the media of infected cells to human C4b. Solid triangles and squares show binding activity of USA+CCP and ROC 2003, respectively, to C4b (solid lines) or to control wells with BSA alone (dashed lines). Open triangles and squares show binding activity of USA and ROCΔCCP, respectively, to C4b. Also graphed is a known concentration of rVCP (star symbol with dashed line) from which we estimated the amount of MPXV CCP in the media of infected cells. The starting amount of rVCP was 2.5 ng (1.75 nM) and was then similarly diluted 2-fold. The error bars in this figure were smaller than the symbols.

We compared the biological activity of the secreted complement control protein from the USA+CCP strain and the ROC 2003 strain, using a C4b binding activity assay (Figure 3). This assay determined the ability of concentrated virus supernatants containing CCP to interact with human C4b coated on a microtiter plate. The monkeypox-infected cell supernatants, from ROC 2003 or USA+CCP infections, manifested equivalent levels of C4b binding (Figure 3). No C4b binding activity was seen in the supernatants from cells infected with either the ROCΔCCP or USA strains of MPXV.

**Figure 4:**
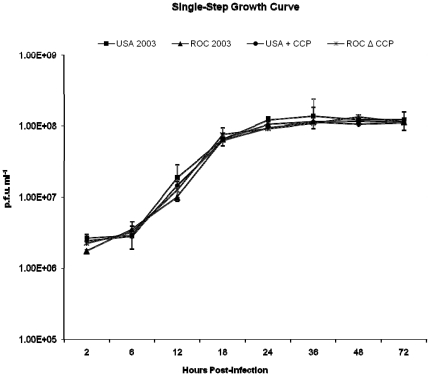
Monkeypox growth curve. Single step growth curves of the parental (ROC 2003, USA 2003) and recombinant (ROCΔCCP, USA+CCP) monkeypox virus strains. No statistically significant differences were identified between the strains (p>0.05).

To further evaluate whether the recombination manipulations affected the biologic properties of the viruses, relative to their growth kinetics, viral one step growth curves were compared. Over 72 hours, no significant differences in growth kinetics were observed between the recombinant and the parental viruses (Figure 4).

### Monkeypox prairie dog challenge

Previous work with black-tailed prairie dogs (*Cynomys ludovicianus*) has demonstrated that monkeypox virus challenge of this species provides an animal model that resembles many aspects of human systemic orthopoxvirus disease [5], [7], [8]. Four challenge groups, each containing four prairie dogs, were inoculated intranasally with 4×10^5^ pfu of ROC 2003, ROCΔCCP, USA 2003 or USA+CCP in a total volume of 10 microliters. This dose was ∼3 times greater than the LD_50_ for the West African strain (1.29×10^5^ pfu) and ∼70 times greater than the LD_50_ for the Congo Basin strain (5.9×10^3^ pfu) in prairie dogs [34]. All animals were observed daily for signs of illness, with weight measurements, swabs, and blood samples taken every other day for the duration of the study (30 days).

### Deletion of CCP in the Congo Basin clade virus slows the disease course and minimizes disease mortality

**Figure 5:**
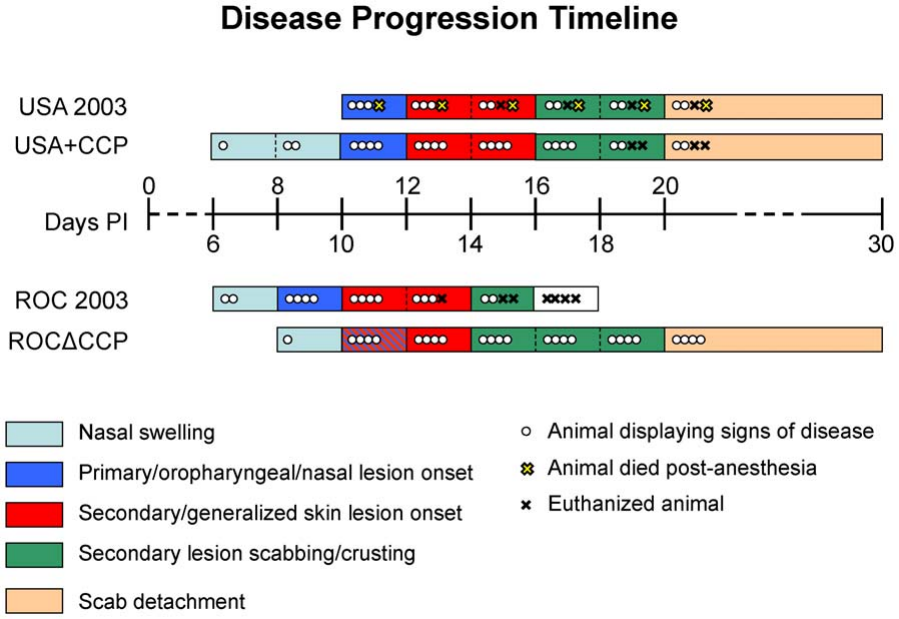
Monkeypox disease progression timeline. Depiction of the comparative disease progression of the four challenge monkeypox strains in the prairie dog animal model. This diagram highlights monkeypox disease as observed lesions progressed from a local to systemic infection and eventual resolution. Note the slower progression of disease in the ROCΔCCP-infected group when compared to its ROC 2003 parental group. The USA+CCP-infected prairie dogs displayed earlier subjective signs of infection when compared to the USA 2003 group.

Similar to previous observations using the intranasal route of infection [8], the first observable presentation of illness was nasal swelling and lesions in the oral cavity (Figure 5). In animals challenged with the recombinant ROCΔCCP, there was a delay in illness onset. ROC 2003 challenged animals manifested nasal swelling at day 6 (2 of 4 animals) and primary lesions (all 4 animals) consisting of several flat, circular macules on the tongue and palate between days 8–10. In contrast, animals challenged with ROCΔCCP manifested nasal swelling later; only one animal showed any nasal swelling at day 8, and primary oropharyngeal lesions did not develop until day 10 (Figure 5, Table 1).

**Table 1:** Comparison of disease signs in the prairie dog challenge study.

Study and disease characteristics	USA 2003		USA+CCP		ROC 2003		ROCΔCCP	
	n = 4*		n = 4		n = 4		n = 4	
**Primary lesion onset**	**P2**	–	**P7**	Day 8	**P8**	Day 8	**P5**	Day 10
	**P4**	Day 10	**P12**	Day 10	**P9**	Day 8	**P11**	Day 10
	**P6**	Day 10	**P14**	Day 10	**P10**	Day 8	**P13**	Day 10
	**P16**	Day 10	**P15**	Day 10	**P18**	Day 8	**P17**	Day 10
**Secondary/generalized lesion onset** †	**P2**	–	**P7**	Day 14	**P8**	Day 10	**P5**	Day 14
	**P4**	Day 14	**P12**	Day 12	**P9**	Day 12	**P11**	Day 12
	**P6**	Day 12	**P14**	Day 14	**P10**	Day 12	**P13**	Day 12
	**P16**	Day 12	**P15**	Day 14	**P18**	Day 10	**P17**	Day 10
**Day 14 secondary lesion count**	**P2**	–	**P7**	6	**P8**	15	**P5**	9
	**P4**	5	**P12**	8	**P9**	13	**P11**	11
	**P6**	8	**P14**	3	**P10**	14	**P13**	10
	**P16**	7	**P15**	7	**P18**	15	**P17**	12
**Objective/subjective signs of disease**	Crusty nose, labored breathing, bloody oral swabs, inappetence, lethargy, weight loss		Crusty nose, labored breathing, bloody oral swabs, inappetence, lethargy, weight loss		Crusty nose, ocular lesions, bloody oral swabs, inappetence, lethargy, severe weight loss, labored breathing		Crusty nose, ocular lesion, bloody oral swabs, inappetence, lethargy	
**Mortality**	50%		50%		100%		0%	
**Day of death/euthanasia (PD#)**	10 (PD2), 14 (PD4)		18 (PD12, PD15)		12 (PD18), 14 (PD10), 16 (PD8, PD9)		–	
**MPXV DNA detected in oral swabs**	Days 2–30		Days 4–26		Days 2 – death		Days 4–30	
**MPXV viable virus in oral swabs**	Days 2–20		Days 4–20		Days 2 – death		Days 4–22	
**Anti-OPXV antibodies**	All surviving animals anti-OPXV positive		All surviving animals anti-OPXV positive		All animals anti-OPXV positive		All animals anti-OPXV positive	

* - Prairie dog PD2 (underlined) died post-anesthesia on day 10.

† - Prairie dogs were observed every other day.

**Figure 6:**
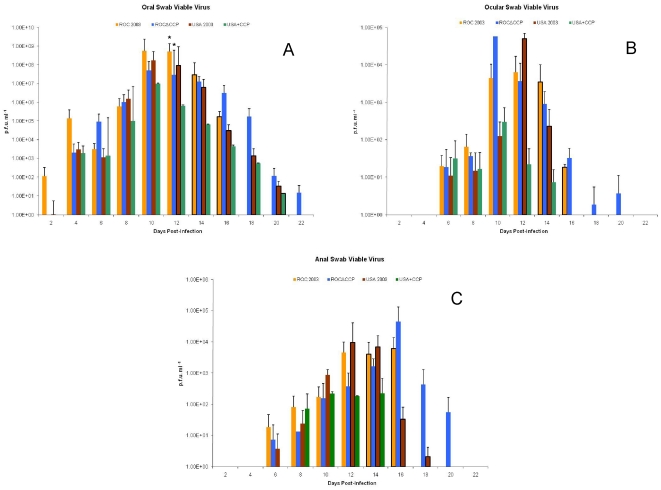
Viable monkeypox virus loads. Mean live virus titers for the oral (A), ocular (B), and anal (C) swabs taken from each animal in a given infection group in the prairie dog challenge study. Note that all animals in the ROC 2003 group were dead by day 16. Also, by day 16 there were only two remaining animals in the USA 2003 group and starting on day 20, there were only two remaining animals in the USA+CCP group. Dark bar borders indicate n<4 for the mean generated on that day. The mean viral titer for each infection group was graphed with standard deviations. Unless indicated by an asterisk, group comparisons within sampling days were not significantly different (p>0.05).

**Figure 7:**
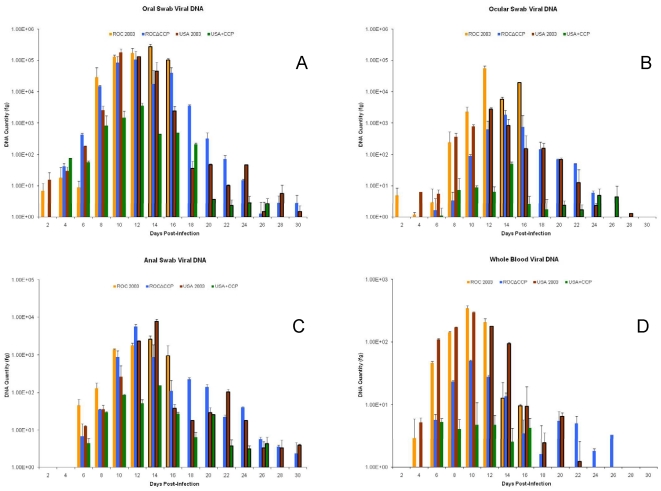
Monkeypox virus DNA loads. Mean viral DNA by real-time PCR for the oral (A), ocular (B), anal (C) and blood (D) samples collected from each animal in a challenge group. Dark bar borders indicate n<4 for the mean generated on that day. The mean viral DNA for each infection group was graphed with standard deviations. Group comparisons within sampling days were not significantly different (p>0.05).

**Figure 8:**
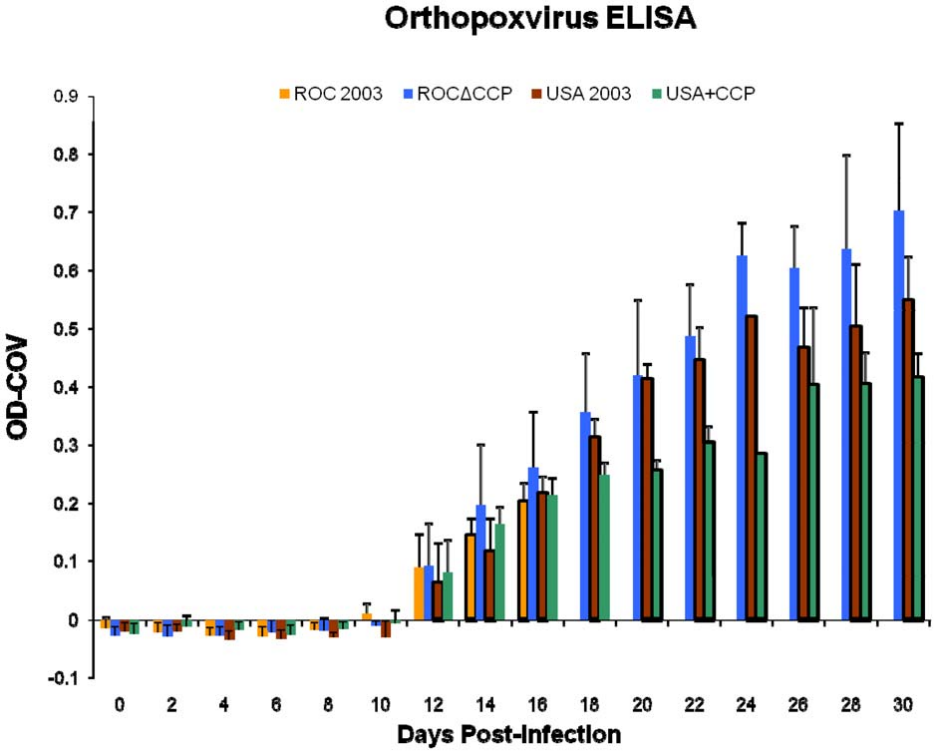
Anti-orthopoxvirus antibody production. Orthopoxvirus specific ELISA to detect total IgG. The average value per infection group is shown here. The reported OD-COV (cutoff value) corresponds to the value above the average signal of the negative control wells plus two standard deviations. None of the average OD-COVs were significantly different between groups within a sample day (p>0.05). Dark bar borders indicate n<4 for the mean generated on that day.

Viable virus and viral DNA was detected in the oral cavity as early as day 2 and remained for several days (Figures 6A, 7A), persisting after the development of orthopoxvirus-specific antibodies beginning on day 12 (Figure 8) in ROC 2003-infected animals. Similar to observation of delayed onset of oropharyngeal lesions, viable virus and viral DNA was detected only after day 4 in ROCΔCCP infected animals (Figures 6A, 7A).

Viral DNA was detected in whole blood of ROC 2003-infected animals at day 4, and not until day 6 in ROCΔCCP infected animals. The overall level of viral DNA in whole blood, on average, was lower in ROCΔCCP infected animals than in ROC 2003-infected animals. (Figure 7D). ROC 2003-infected animals developed a disseminated, vesiculo-pustular rash between days 10–12, with average lesion sizes measuring 5 mm in diameter appearing on the back, abdomen, armpit and hindleg regions of the prairie dogs (Table 1). The ROC 2003-infected prairie dogs developed secondary lesions on multiple sites, such as the back, armpits, and abdominal area; lesions were also observed on the footpads of all prairie dogs. In some animals, pustules developed on the lip and edges of the eyelids, and lesions were found on the genitals of two male animals. In most ROCΔCCP infected animals, secondary or generalized lesion onset was delayed with respect to the observations within the ROC-infected cohort, although in one ROCΔCCP infected animal (PD17) we observed the appearance of a secondary lesion on its neck on the same day as its first oral lesion (day 10). Secondary lesions were found in the other ROCΔCCP-infected prairie dogs between days 12–14 (Figure 5, Table 1), with a similar lesion distribution as seen in the ROC 2003-infected animals, but the lesion load was less than what was documented in the ROC 2003-infected animals (Table 1).

**Figure 9:**
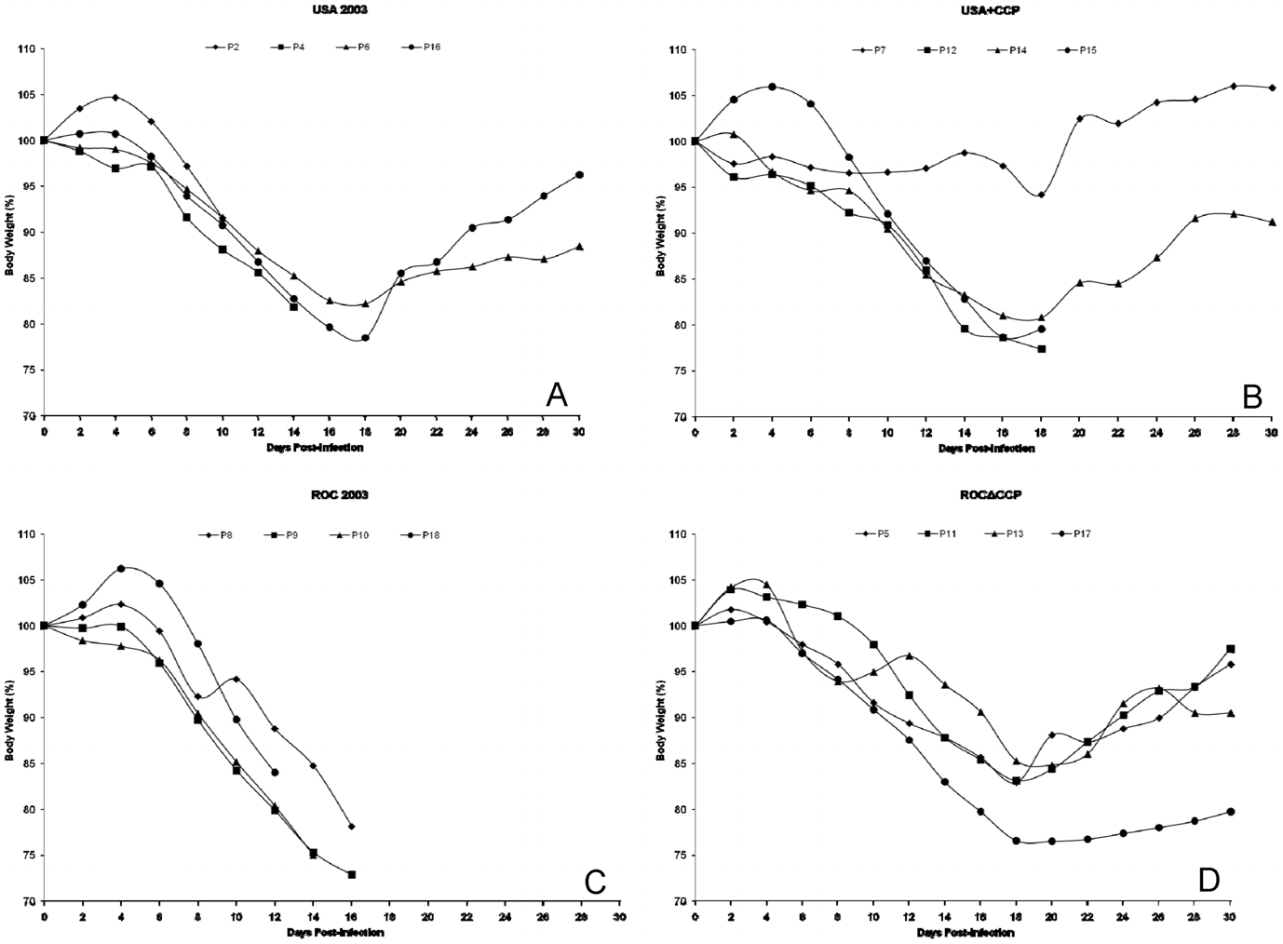
Prairie dog weight graphs. Percent weight loss graph for all four infection groups, USA 2003 (A), USA+CCP (B), ROC 2003 (C), and ROCΔCCP (D). Each prairie dog within their respective infection group was listed with a different symbol according to legend.

Twelve days post-infection it was evident that weight loss in the ROC 2003 group was occurring more rapidly and at a greater magnitude than any of the other challenge groups with 17% weight loss vs 8% for ROCΔCCP, 13% for USA 2003 and 11% for USA+CCP on this day (Figure 9). One animal was euthanized on this day (PD18) due to inappetence, lesion severity, lethargy, and labored breathing. The condition/health of the remaining animals in this group rapidly declined as an additional prairie dog was euthanized on day 14 (PD10) and the remaining two animals were euthanized by day 16, due to a combination of disease symptoms such as lethargy, labored breathing, and inappetence including >25% weight loss for PD9 (Figure 9, Table 1). Both viable virus and viral DNA were first detected on day 2 and persisted until the death of each animal (Figures 6 & 7, Table 1). Upon necropsy, viable virus was detected at varying levels in all sampled tissues with peak amounts found within the kidney for all four animals (Table 2).

**Table 2:** Viable virus in animal necropsy tissue samples in plaque-forming unit per gram of tissue.

Strain	Animal Number	Death/Euthanasia	Heart	Lungs	Spleen	Kidney	Liver	Lymph node	Gonad	Eyelid
**ROC 2003**	**PD8**	**16**	50,331	410,048	178,922	495,522	1,110	389	19,343	18,485
	**PD9**	**16**	34,258	94,949	391,403	2,012,146	2,083	1,131	22,448	6,510
	**PD10**	**14**	124,812	960,938	1,138,393	2,586,957	31,154	24,258	203,284	898,785
	**PD18**	**12**	317,588	359,512	430,380	953,737	9,785	704	160,887	284,211
**ROCΔCCP**	**PD5**	**30**	0	0	0	0	0	0	0	0
	**PD11**	**30**	0	0	0	0	0	0	0	0
	**PD13**	**30**	0	0	0	0	0	0	0	0
	**PD17**	**30**	0	0	0	0	0	0	0	0
**USA 2003**	**PD2** *	**10**	586	383	671	0	0	0	0	11,856
	**PD4**	**14**	19,704	5,865	39,488	8,347	0	0	0	0
	**PD6**	**30**	0	0	0	0	0	0	0	0
	**PD16**	**30**	0	0	0	0	0	0	0	0
**USA+CCP**	**PD7**	**30**	0	0	0	0	0	0	0	0
	**PD12**	**18**	0	8,651	21,073	33,879	8,916	0	0	0
	**PD14**	**30**	0	0	0	0	0	0	0	0
	**PD15**	**18**	0	19,335	5,670	13,158	14,431	0	0	0

* Death due to anesthesia.

Conversely, the ROCΔCCP-infected cohort demonstrated resolution of disease; weight gain reinitiated between days 18 and 20, by day 16 all lesions had scabbed over, and by day 22 most of the crusts had fallen off, leaving hypopigmented scars on the skin. Prior to euthanasia of the ROC-infected animals, there were no significant differences between the viable viral loads in oral, ocular or anal excreta of ROCΔCCP-infected and ROC 2003-infected animals on any day of the study, except for oral swabs taken on day 12 post-infection (p = 0.03) (Figure 6A, B, & C). On this particular sample day, the ROCΔCCP-infected cohort average viable virus load was lower than that of the wild-type ROC-infected cohort. The average oral and ocular shedding of viable ROCΔCCP virus peaked day 10, and consistently decreased until it was undetectable after day 22 (Figure 6A and 6B). Anal swabs detected viable ROCΔCCP through day 20 with peak average shedding occurring on day 16. The duration of viral shedding cannot be compared to the wild-type ROC 2003-infected animals since these succumbed to disease by day 16 post-infection, however the ROCΔCCP-infected animals demonstrated shedding of viable virus longer than seen for the USA-infected animals (Table 1 and Figure 6). The ROCΔCCP-infected cohort displayed slower weight loss than the wild-type ROC-infected cohort. Linear regression analysis of the average weight loss data from the highest to the lowest weight indicated that the slope of the ROC 2003-infected animals was significantly higher than in ROCΔCCP group (p = 0.03). The analysis also showed that the average weight loss in the ROCΔCCP group was not significantly different from the USA 2003 group (p = 0.69). These findings highlighted the fact that on average the ROC 2003-infected animals displayed signs of greater disease severity (such as rapid weight loss) than the ROCΔCCP-infected prairie dogs.

Although not statistically significant, all ROCΔCCP-infected prairie dogs seroconverted to the highest level of any of the four groups in the challenge study (Figure 8). At necropsy, no viable virus was found in any of the tissues from the ROCΔCCP-infected animals (Table 2) but weak positives for viral DNA were detected in the following tissues of at least two animals in this group: lungs, heart and kidney (data not shown).

### Addition of CCP in the West African clade virus does not markedly accelerate clinical disease course, variably affects virus shedding and does not affect disease mortality

Prairie dogs infected with USA 2003 did not show any signs of infection until day 10 when the first macules appeared on the tongues and palates of 2 animals (PD4 and PD16) (Figure 5). Only animals in the USA+CCP group exhibited the early symptom of nasal swelling on day 6 (PD12) and on day 8 (PD7) (Figure 5). Macular lesions appeared on the tongue or palate in all animals on day 10, similar to that observed with the USA 2003-infected prairie dogs.

Within the USA 2003 infected group, on day 10, one of the prairie dogs (PD2) in this group did not wake up post-anesthesia. PD2 did not demonstrate lesion development or other overt signs of disease on day 10, and did not have significant weight loss (<10%, Figure 9), and had very low viable virus levels in tissues upon necropsy (Table 2), suggesting its death most likely was due to complications with anesthesia and not related to monkeypox infection.

Unexpectedly, viable virus was detected relatively early (Day 2) in the oral swab of one USA 2003-infected animal (PD16), and in others by day 4. Viral excreta in ocular or anal swabs of animals infected with USA 2003 were not observed until day 6 and persisted in surviving animals out to day 14 for ocular swabs and day 18 for anal swabs (Figure 6). Viral DNA persisted longer than viable virus in oral, ocular and anal excreta samples (Table 1, Figures 6 and 7). Viral DNA was detected within the blood as early as 4 days post-infection and persisted until 22 days post-infection (Figure 7D). Animals challenged with the USA+CCP virus began to excrete virus in the oral, ocular and anal samplings on days 4, 6 and 8 respectively. After day 10, measured viable virus and DNA loads were consistently lower than that observed in the USA 2003-infected animals in the oral, ocular and anal swabs with the only exception being on a day 18 DNA oral swab (Figures 6 and 7). Viral DNA was detected in whole blood on day 6, persisted through day 16 and levels were less than that observed in USA 2003 infected animals throughout the study (Figure 7D). However, none of these differences in viable virus or viral DNA load were statistically significant.

Secondary or generalized lesions (1–3) appeared between days 12 and 14 on the anus of all USA 2003-infected animals and the genitals of two male prairie dogs. On day 14, PD4 was euthanized due to inappetence, lethargy, and labored breathing (Figure 5, Table 1). By day 16 all observed lesions became umbilicated and began to scab over; by day 24 most of the lesions had resolved leaving patches of hypopigmentated skin. As seen in the USA 2003-infected group, the USA+CCP-infected animals did not develop many secondary lesions, a few were found in the mouth or around the anus and these lesions were first identified on day 12 (Table 1). On day 18, two of the animals (PD12 and PD15) were euthanized due to lethargy, inappetence and labored breathing, along with weight loss near the euthanasia limit (Figure 9B). The rate of weight loss was similar for both the wild-type USA 2003-infected cohort and the USA+CCP-infected cohort. Linear regression analysis of the average weight loss data from the highest to the lowest weight showed no significant difference between the slopes of USA 2003 and USA+CCP-infected animals (p = 0.49).

Upon necropsy of the USA 2003-infected animals, viable virus was detected in the lungs, heart, kidney and spleen of PD4 who was euthanized on day 14, while in PD2, the animal who did not revive after anesthesia on day 10, viable virus was detected in the heart, lung, spleen and eyelid samples (Table 2). At necropsy, no viable virus was found in any of the tissues from the animals who survived the viral challenge, although low levels of viral DNA was present in the oral swabs of PD6 and PD16 (Figure 7).

Upon necropsy of the USA+CCP-infected animals that were euthanized on day 18 post-challenge (PD12 and PD15), viable virus was recovered from the lungs, kidney, liver and spleen. Viable virus was found in the liver of USA+CCP or ROC2003-infected animals who did not survive to day 30, but was not found in the liver of USA2003-infected PD4, which was euthanized on day 14 of the challenge study. At the day 30 necropsy of the survivors of the USA+CCP infection, no viable virus was found in any of the tissues, but there was a weak positive DNA signal detected in the eyelid sample of one animal (PD6).

## Discussion

The goal of this study was to determine if the proposed immunomodulatory effect of the monkeypox CCP was a major contributor to the documented disease differences between the West African and Congo Basin clades of monkeypox virus. To investigate the role of CCP in monkeypox virulence, we deleted it from the more virulent ROC 2003, a Congo Basin strain, and incorporated it into the less virulent USA 2003, belonging to the West African clade. The selectable marker, *gpt*, has been used in the creation of recombinants for *in vivo* studies successfully but its presence in the recombinants has the potential to be a limitation in this study, hence we conducted a C4b assay to confirm normal CCP activity and growth curve to demonstrate normal growth kinetics of the recombinants. The experimental design was discussed with multiple institutional ethics oversight committees prior to its execution. It was important to investigate the addition of CCP to the USA 2003 strain to better frame the scientific question regarding disease manifestations influenced by the presence or absence of CCP within these strains of monkeypox viruses. Due to several gene truncations/deletions in the West African strain, we sought to determine if the addition of a single virulence-associated gene was sufficient to impact its pathogenesis. We confirmed by single-step growth curves that the mutations introduced in the recombinant viruses had no effect on the *in vitro* infectivity and growth of the virus. Using a C4b-binding ELISA, we confirmed that the CCP produced by the USA+CCP had the same activity as the CCP naturally expressed by ROC 2003. Liszewski, et al. reported that recombinant forms of VCP (rVCP) and the MPXV CCP had approximately the same C4b binding activity [9]. Using the rVCP as a standard and the concentrated supernatants from MPXV-infected cells, we estimated that the concentration of CCP in the media of infected cells to be ∼0.12 µg/ml. This estimation assumes that the activity or the protein in the supernatant of infected cells is similar to purified recombinant proteins. Through the Ara-C experiment, we also provided evidence that VCP and CCP were proteins expressed at the late stages of infection. VACV-infected cells produced more VCP than monkeypox-infected cells produce of its homologue (Figure 2A).

The parental and recombinant strains of virus were used in the prairie dog animal model, which has proven to be a reproducible model capable of distinguishing disease differences between the two clades of monkeypox virus [5]. The intranasal route of infection mimics an upper respiratory exposure, which is an important route of transmission [35]. Similar to intradermal infections of mice, guinea pigs, and rabbits with VCP-knockout virus [20], [26], [28], the ROCΔCCP group of infected animals displayed a marked reduction in disease severity, and in this study they produced fewer disseminated lesions with no mortality. The linear regression analysis of the weight loss data further highlights the reduced disease severity seen in the ROCΔCCP-infected group when compared to the wild-type ROC 2003-infected group. The loss of CCP significantly reduced the rate of weight loss compared to ROC 2003-infected group (p = 0.03) and made the weight loss kinetics more closely resemble that observed in USA 2003-infected animals (p = 0.69). The only other difference in the rate of weight loss that neared 95% statistical significance was between wild-type ROC 2003-infected and wild-type USA 2003-infected groups (p = 0.057). The ROCΔCCP-infected group also appeared to have delayed signs of disease, with the onset of nasal swelling and primary lesions beginning later than the ROC 2003-infected group (Figure 5). ROCΔCCP-infected animals excreted similar quantities of virus (Figure 6) at “peak” times post-infection when compared to ROC 2003 despite less severe disease with lower rash burden (Table 1). Detection of viral DNA in the blood of ROCΔCCP-infected animals was delayed and reduced with respect to parental ROC 2003-infected animals (Figure 7), perhaps indicating that the loss of CCP reduced the ability to suppress elements of the innate response, enabling the hosts to localize the infection, thus increasing time until systemic viral disease.

The ROCΔCCP recombinant-infected prairie dogs elicited the strongest humoral immune response of all monkeypox virus strains tested (Figure 8). Since all of the ROC 2003 animals succumbed to disease by day 16, we were unable to compare the magnitude of humoral immune response, persistence of viable virus and possibility for shedding/transmission between the ROC 2003 and ROCΔCCP groups. However, consistent with previous studies [8], the duration of viable virus excretion/viremia for the ROCΔCCP-infected animals was greater than that seen for USA 2003-infected animals, indicating other factors besides CCP affect the overall potential for shedding (and thus potentially transmission) of the virus. Overall, the ROCΔCCP-infected prairie dogs were able to avoid the severe morbidity, increased mortality and increased weight loss associated with the Congo Basin infection and instead manifested a disease progression more similar to the USA 2003-infected animals (Figures 5, 6 & 9). The data presented here demonstrates that the monkeypox CCP homologue is a significant immunomodulatory protein for Congo Basin clade virulence. Our data also suggests that deletion of CCP from ROC 2003 renders the virus susceptible to clearance by the innate and adaptive immune system, and permits a robust adaptive immune response to virus infection.

Notably, the inclusion of CCP in the West African clade monkeypox virus did not enhance its virulence to levels comparable to that of the Congo Basin clade viruses, but appeared to accelerate development of some signs of the disease course. Overall, the mortality and morbidity associated with the USA 2003+CCP recombinant infections were within expected ranges estimated by the LD_50_ for the USA 2003-infected animals [34]. When compared to the USA 2003-infected animals, there did appear to be some earlier signs of localized, “primary” disease, such as the observation of nasal swelling in PD12 (day 6, 8) and PD7 (day 8), the progression of disease in this regard is similar to the ROC 2003-infected group (Figure 5). Other than earlier nasal involvement, all other later, generalized signs of disease remained unchanged from the USA 2003 group; for instance there was not an appreciable increase in the onset of, or number of, lesions (Table 1). Furthermore, the duration of viable virus shedding in oral swabs was almost indistinguishable (Figure 6), providing greater evidence that the presence of CCP does not influence the potential for shedding/transmission of the virus. The presence of CCP may affect the tissue tropism and pathogenesis, the virus was found in the liver only in euthanized animals infected with either USA+CCP or ROC 2003 (Table 2). This remains a preliminary observation as none of the ROCΔCCP infected animals were euthanized during the disease course.

The prairie dog model system is useful because of the similarity to human systemic orthopoxvirus disease, and potential extrapolations to understanding human disease. However, the use of outbred animals does make experimental designs to achieve statistical significance, if outcome differences are subtle, challenging. For instance, in order to detect a significant difference (p<0.05) in the linear regression slopes of the weight loss in the USA 2003 vs. USA+CCP groups, we would have to reach a statistical power of 80% which would require 67 animals per group.

Our data indicates other factors besides CCP are necessary to significantly increase disease virulence. Although there are differences, this is a conclusion also reached by another group when testing a CCP knockout monkeypox virus recombinant in the non-human primate model [36]. Homologues to several other virulence factors such as the myxoma virus M-T4, anti-apoptosis protein and B14R, interleukin-1 binding protein are also missing from the West African clade genomes and are inferred to be involved in determining disease pathogenesis and virulence [5]. However, our data with the West African clade parental and recombinant viruses additionally suggests that the presence of CCP may have an effect on the host adaptive immune response as antibody levels were lower in the two surviving USA+CCP-infected animals versus the two surviving USA 2003-infected animals (Figure 8).

These results also highlight CCP's importance as a potential target for immunotherapy. A strain of vaccinia virus lacking VCP, yet possessing all the other major antigenic epitopes, may be a promising vaccine due to its reduced virulence and potential to elicit a stronger immune response. Others have described the use of monoclonal antibodies (mAbs) to block the activity of the CCP homolog, SPICE, found in variola virus [37]. Monoclonal antibodies directed against CCP [25], [37] and possibly several other established virulence-related proteins [38] may prove to be effective in treating infected individuals.

Finally, an observation of this study which may impact the use of viral DNA measurements to track disease course and the effect of disease interventions was that all sampling group measurements of viral DNA had similar kinetics to that of viable virus. However, viral DNA persisted much longer than viable virus within the samples (Figures 6 and 7). The RT-PCR assay is likely to detect DNA fragments as well as genomic DNA belonging to intact viral particles, thereby viral DNA observed later during infection may be due to the clearing of the infection. A better understanding of what the presence of viral DNA signifies will be critical to determining its utility as a biomarker for studies evaluating vaccine or therapeutic efficacy.

This study demonstrates CCP expressed by ROC 2003, an example of the Congo Basin clade of monkeypox virus, is a virulence factor that significantly affects clinical disease progression, weight loss and mortality in the prairie dog animal model system. However, its expression in the West African clade virus, represented by USA 2003, is insufficient to elicit equivalent levels of disease comparable to the Congo Basin clade in this model system. These viruses have divergent evolutions [4], possibly due to different reservoir or susceptible host species, and thus may have developed different virulence mechanisms. Regardless, removal of CCP from the Congo Basin virus clade significantly reduced the morbidity and mortality associated with disease.

## Materials and Methods

### Parental Viruses and Cell lines

The West African strain of monkeypox virus used for these experiments was MPXV-USA-2003-044 (USA 2003). This isolate was collected during the 2003 monkeypox outbreak in the USA from the lymph node of an infected prairie dog associated with the initial human case in Wisconsin [4]. The Congo Basin strain of monkeypox virus, MPXV-ROC-2003-358 (ROC 2003), was isolated from the rash of a 10-year-old girl infected in Impfondo, Republic of Congo, and admitted to the hospital on June 9, 2003 [39]. Both monkeypox strains were passaged three times prior to working stock production. Supernatant containing VCP produced by low-passage, Vaccinia virus Western Reserve (VACV WR) was utilized for comparisons to monkeypox CCP. BSC-40 cells [40], grown in RPMI 1640 containing 10% sterile-filtered, fetal bovine serum (FBS) were used for all cell culture infections and isolations of all parental and recombinant virus strains, unless otherwise specified.

### Recombinant *gpt* ^+^ Virus Construction

All work was approved by the Institutional Biosafety Committee and the Select Agent office prior to the initiation of these experiments. The selectable genetic marker used for isolation was the *Escherichia coli* xanthine-guanine phosphoribosyltransferase or *gpt* gene, which confers resistance to growth inhibitor, mycophenolic acid [41], [42].

The CCP gene plus 123 bp of the upstream region was PCR amplified from ROC 2003 with primers CCP1, 5′-GTC GAC CTC ATA AGT C AC TGC CAT TGT TTT-3′ and CCP2, 5′-GGA TCC CAT TGT AGT TGT ATG AGT GT A TG-3′ using Platinum High Fidelity DNA Polymerase (Invitrogen, Carlsbad, CA), then ligated into the pZsGreen expression vector at the *Sal* I and *Bam* HI sites for the construction of pCDgpt. This insert was positioned into plasmid pCDgpt (Figure 1) adjacent to the *gpt* gene under the control of a vaccinia-derived, synthetic early-late promoter, 5′-GCA TAT GTA AAA GTT GAA AAT ATA TTT CTA TGC TAT AAA TA-3′ [43] and flanked by sequences homologous to the 765 bp non-coding intergenic region between ORFs 176 and 177 (annotated as type I membrane hemagglutinin and serine/threonine kinase, respectively) in the USA 2003 strain. Plasmid pABgpt (Figure 1) contained the synthetic early-late promoter controlling the *gpt* gene with upstream and downstream flanking regions homologous to ORFs 21 and 23 (annotated as unknown protein and kelch-like gene fragment, respectively) from the ROC 2003 genome.

The two plasmid constructs were integrated into their host genomes through an infection-transfection protocol. BSC-40 cells in RPMI+2% FBS are seeded in 6-well Costar plates (Corning Inc., Corning, NY) 24 hours prior to the experiment, with an average confluency of ∼80% per well. The cells are washed in Opti-MEM media, then either ROC 2003 or USA 2003 sucrose cushion-purified virus was added to each well at a MOI = 0.05. The viruses were incubated at 36°C, 6% CO_2_ for 2 hours, and then replaced with Lipofectamine 2000 (Invitrogen) in Opti-MEM and 4 µg of the plasmid DNA of interest (pABgpt or pCDgpt) for 72 hours.

Virus plaques were purified using mycophenolic acid as the selection agent [41], [42]. The recombinant viruses successfully expressing xanthine-guanine phosphoribosyl-transferase were selected by growth in BSC-40 cells in 2X DMEM supplemented with 2% FBS, 25 µg/ml mycophenolic acid, 250 µg/ml xanthine, 10 µg/ml thymidine, 15 µg/ml hypoxanthine and 2 µg/ml aminopterin (Millipore, Phillipsburg, NJ). Each recombinant strain was plaque purified four times in a 25 µg/ml mycophenolic acid plus 2% agarose overlay containing 3% neutral red, and collected using a Pasteur pipet after incubation at 36°C, 6% CO_2_ for 72 hours.

The West African recombinant strain of monkeypox virus, MPXV-USA-2003-044, in which CCP was incorporated into its genome through homologous recombination, was designated USA+CCP. The CCP knockout recombinant derived from the Central African strain, MPXV-ROC-2003-358, was designated ROCΔCCP. Each recombinant clone was sequenced using 454 sequencing in the CDC Biotechnology Core Facility to ensure the genomes were free of second site mutations.

### Complement control protein expression

BSC-40 cells were infected with VACV WR, ROC 2003, USA 2003, ROCΔCCP, or USA+CCP virus, at a MOI = 10 in Opti-MEM at 36°C, 6% CO_2_ for 1 hour. The media was removed and replaced with Opti-MEM (Invitrogen, Carlsbad, CA) then incubated for 24 hours at 36°C and 6% CO_2_. The supernatants were decanted into a centrifuge tube and then spun at 1000× g for 10 min to remove cell debris. After UV inactivation for 2 min. at 25°C, all harvested supernatants were subjected to ∼5-fold concentration (by volume) using the Ultracel YM-10 Centricon protein concentration columns (Millipore, Phillipsburg, NJ) with a 10 kDa molecular weight cut-off. Centricons were spun at 5000× g for 10 min at 25°C and filtrate discarded, retentate was collected at 1000× g for 2 min at 25°C. To harvest the cell lysates, the infected-cell monolayers were washed twice with Opti-MEM, and then RIPA buffer was added to the cells, which were then incubated at 4°C for 5 min (Thermo Scientific, Rockford, IL). The cells were collected and spun at 14,000× g for 15 min at 4°C.

### Western blot analysis

For Western blot analysis, the above virus-infected cell supernatants and cell lysate samples were boiled for 5 min. in Laemmli loading buffer containing 2% sodium dodecyl sulfate and 2-mercaptoethanol (Bio-Rad Laboratories, Hercules, CA). All supernatant and cell lysate samples (3 µg/ml per well) were loaded onto a 12% precast Tris-glycine polyacrylamide gel (Bio-Rad Laboratories, Hercules, CA) and then run at 150 volts for 1 hour. Protein bands were transferred at 100 mA for 50 min to polyvinylidene fluoride (PVDF) membranes using a Mini-blot protean II chamber (Bio-Rad Laboratories, Hercules, CA). The membranes were incubated in blocking buffer (1% non-fat milk, 0.1% Tween 20) for 2 hours. The blots were probed with polyclonal rabbit anti-VCP antibody (courtesy of Dr. John Lambris, University of Pennsylvania) at a 1∶1000 concentration for approximately 12 hours on a 10 rpm rocker at 4°C. The blots were also probed with polyclonal rabbit anti-transferrin at 1∶1000 to confirm to that wells received an equivalent load of protein. After the blot was washed with PBS containing 0.1% Tween 20, alkaline phosphatase conjugated goat anti-rabbit antibody (1∶3000) (Bio-Rad Laboratories, Hercules, CA) was added to the blot for 2 hours and the chemiluminescent signal detected using ChemiDoc XRS+ (Bio-Rad Laboratories, Hercules, CA).

### Expression of the complement control protein

BSC-40 cells were infected with VACV WR or ROC 2003 at MOI = 10 in Opti-MEM with or without 10 mg/ml cytosine arabinoside [Ara-C] (Sigma-Aldrich, St. Louis, MO) at 36°C, 6% CO_2_ for 1 hour. The media was aspirated, cells washed twice with 5 ml of the appropriate media, and then 7 ml of the corresponding media was added to each flask and incubated at 36°C, 6% CO_2_. Infected cell supernatants were collected at 2, 4, 6, 10, 24 and 36 hours post-infection (hpi) and replaced with fresh corresponding media after each harvest (Figure 2C). Ultracel YM-10 Centricon protein concentration columns were used to concentrate the harvested vaccinia and monkeypox supernatants. The samples were spun at 5000× g for 10 min at 25°C and filtrate discarded, retentate was collected at 1000× g for 2 min at 25°C. Samples were analyzed by Western blot as described above.

To compare the temporal expression of CCP between ROC 2003 and USA+CCP strains, we repeated the above experiment using those strains and VACV WR as a control in the presence/absence of 10 mg/ml Ara-C. Infected cell supernatants were harvested at 24 hours post-infection, then the vaccinia and monkeypox supernatants were ∼5X concentrated by Centricon, and all samples were analyzed by Western blot (Figure 2D).

Band intensities were measured using the Image Lab 3.0 software band density tool (Bio-Rad Laboratories, Hercules, CA).

### Complement control protein C4b binding assay

C4b ELISAs were performed as described in the literature [9]. Briefly, Maxisorb ELISA plates (Nunc, Rochester, NY) were coated with human C4b (Complement Technologies, Tyler, Texas) at 5 µg/ml in PBS overnight at 4°C and then blocked for 2 hours at 37°C (5% BSA (Sigma A2934) and 0.1% Tween 20 in PBS). Concentrated supernatants (∼5X) from cells infected with ROC 2003, ROCΔCCP, USA 2003, or USA+CCP were serially diluted in low salt ELISA buffer (10 mM Tris (pH 7.2), 25 mM sodium chloride, 0.05% Tween 20, 4% BSA, and 0.25% Nonidet-P40), added to wells in duplicate, and incubated for 1 hour at 37°C. Plates were then washed in low salt ELISA buffer (but not containing BSA), and rabbit anti-VCP (diluted 1∶5000 in low salt ELISA buffer) was added for 1 hour at 37°C. Following washing, a peroxidase-conjugated donkey anti-rabbit IgG (diluted 1∶8000 in low salt ELISA buffer) was added for 1 hour at 37°C. Plates were developed using TMB substrate (Invitrogen) and read at an OD of 450 nm. Data shown is the average of two experiments (Figure 3). A standard curve using rVCP (starting amount 2.5 ng) [9] (courtesy of Dr. John Atkinson, Washington University of School of Medicine) was also run in this assay to estimate the amount of MPXV CCP in the supernatant of infected cells.

### Single Step Growth Curve

Confluent monolayers (>95%) of BSC-40 cells in six-well plates were infected, in duplicate, with a MOI = 5 of ROC 2003, USA 2003, ROCΔCCP, or USA+CCP virus in RPMI 1640+2% FBS then incubated at 36°C and 6% CO_2_ for 1 hour. The inoculum was removed and replaced with 1 mL fresh RPMI 1640+2% FBS media. Virus was harvested from the cells at 2, 6, 12, 18, 24, 36, 48, and 72 hours post-infection and stored at −70°C. On day of titration, the aliquots were freeze-thawed 3 times prior to serially diluting the samples and plating on six-well plates of BSC-40 cells. After 72 hour incubation, plates were stained with crystal violet+2% formalin and individual plaques were counted (Figure 4).

### Prairie dog challenge experiment

All animal work was conducted according to relevant national and international guidelines. The experimental designs and ethics of the study were discussed with the institutional biosafety committee, the institutional animal care and use committee, and the select agent program. Animals were cared for in accordance with the CDC Institutional Animal Care and Use Committee (IACUC) approved protocol 1431REGPRAC-A4. Wild-caught, juvenile black-tailed prairie dogs (*Cynomys ludovicianus*) were obtained from western Kansas. The animals were captured shortly after adolescence in 2004 and at the time of infection the prairie dogs had been in captivity for 4 years. During experimental infections, animals were housed individually in 12.13″×23.38″×9.00″ cages with aerosol filter tops (Thoren Caging Systems, Hazleton, PA). Cages were kept in a Duo-Flow biosafety cabinet (Lab Products, Inc. Seaford, DE) in the BSL-3 animal room. Animal handling was performed using BSL-3 personal protective equipment. Throughout the study, animals were maintained in a 12 hour light/12 hour dark cycle, and in addition to standard prairie dog chow (Exotic Nutrition Pet Co., Newport News, VA), the animals were provided with monkey biscuits (Exotic Nutrition Pet Co., Newport News, VA) for dietary enrichment.

Prairie dogs weighing between 875–1250 grams were infected intranasally with 4×10^5^ pfu of ROC 2003, USA 2003, ROCΔCCP, or USA+CCP while under inhaled anesthesia (5% isoflurane mixed with air). There were 4 animals in each of the monkeypox virus-challenged groups. The intranasal inoculum, titered at the time of challenge, was administered in a total volume of 10 µl 1× PBS, with each nostril receiving 5 µl of inoculum. Every 2 days swabs were taken from the mouth, anus and eye of each prairie dog under isoflurane anesthesia using sterile individual Dacron swabs (Epicentre Biotechnologies, Miami, FL) and stored at −70°C without diluent. In addition, 200 µl of whole blood was collected from the lateral saphenous or jugular vein in EDTA coated tubes (Vetlab Supply Inc., Miami, FL) for real-time PCR and viable virus assessment of total viral load; 100 µl was harvested in serum separating tubes (Becton, Dickinson and Co., Franklin Lakes, NJ) for serological analysis. On these sample days the weight and temperature of each prairie dog was recorded and tracked throughout the study. Any notable visual observations or occurrences were also documented on sampling days. For euthanasia criteria, we observed individual animals for the following signs of illness severity: inappetence (3 pts), labored breathing (5 pts), unsteady gate (3 pts), lack of responsiveness to touch (5 pts), and lethargy (3 pts). Points were given for each of these signs, any animal accruing a score indicative of severe illness (≥10 pts.) was humanely euthanized. In addition, any animal losing greater than 25% of their starting body weight was euthanized regardless of other clinical symptoms.

The planned length for this study was 30 days; if death did not occur prior to the end of the challenge experiment, animals were humanely euthanized and necropsied. Necropsies on all animals were performed according to IACUC standards in a BSL-3 laboratory and utilizing full BSL-3 personal protective equipment. Samples taken during necropsy, not including the swab and blood samples, were from the following sites: eyelid, heart, lung, liver, spleen, kidney, mesenteric lymph node, and gonad. Instruments (scalpel, scissors, forceps, etc.) were cleaned and decontaminated with 30% Lysol (Reckitt Benckiser Inc., Parsippany, NJ) and 10% bleach (Fisher Scientific, Pittsburgh, PA) between collections of each tissue. Tissues were frozen at −70°C prior to further processing.

### Sample Processing

All sample processing took place in the BSL-2 laboratory with BSL-3 practices. In order to analyze viral DNA in the blood, water was added as needed to bring the total volume to 200 µl, and the samples were incubated at 55°C for 1 hour. The EZ-1 DNA extraction robot (Qiagen, Valencia, CA) was used for viral DNA extraction of all collected blood samples using the tissue extraction protocol provided by the manufacturer. For all swabs collected during the challenge study, 400 µl PBS was added to the microfuge tube, incubated at room temperature for ∼5 minutes and then the swab extraction tube systems (SETS) (Roche, Indianapolis, IN) protocol was used to recover sample from the swab. A 100 µl aliquot of swab eluant was incubated in lysis buffer and proteinase K at 55°C for one hour prior to extraction of genomic DNA using the EZ-1 DNA extraction robot (Qiagen, Valencia, CA). For all virus strains, the remaining swab lysate was used for virus isolation. There was no viable virus isolation from the prairie dog whole blood samples due to technical difficulties.

Necropsy tissue samples were homogenized to slurry using a metal ball tissue grinder (SPEX SamplePrep LLC, Metuchen, NJ). Genomic DNA was prepared from a 100 µl slurry aliquot using the EZ-1 DNA extraction robot, after incubation in lysis buffer and proteinase K for 1 hour as described above. Remaining necropsy tissue sample slurry was used for virus isolation.

### Real-time PCR analysis

All swab, blood, and necropsy samples were analyzed in duplicate by real-time PCR using forward and reverse primers and probe complementary to the highly conserved orthopoxvirus DNA polymerase gene, *E9L* [8], [44].

### Virus titration

Swab and necropsy samples that tested positive for the presence of orthopoxvirus DNA using the real-time PCR assay were evaluated for the presence of viable virus through growth in BSC-40 cells. These samples were titrated by tenfold dilution of the swab eluent/tissue slurry in RPMI+2% FBS media and incubated with the cells at 36°C and 6% CO_2_ for 72 hours. Cells were stained with crystal violet+2% formalin and then the plaques were counted.

### Serological analysis

ELISA assays were used to analyze the collected blood for the presence of anti-orthopoxvirus-specific IgA and IgG. Ninety-six well microtiter plates were coated with 0.01 µg/well crude vaccinia virus-BSC40 cell lysate in carbonate buffer on one half of the plate and an equivalent volume of BSC-40 cell material diluted in carbonate buffer on the other half; plates were incubated overnight at 4°C. The plates were inactivated by incubating in 10% buffered formalin at room temperature for 10 min., after which they were blocked for 30 min at room temperature with assay diluent (PBS, 0.01 M, pH 7.4+0.05% Tween-20, 5% dried skim milk, 2% normal goat serum and 2% BSA) and washed in PBS plus 0.05% Tween-20. Experimental prairie dog sera (1∶100) was added to both halves of the plates and incubated for 1 hour at 37°C. Each plate was washed in PBST and ImmunoPure A/G conjugate (Pierce, Rockford, IL) was added at 1∶30,000 and then incubated for 1 hour at 37°C. Plates were then washed and a peroxidase substrate (Kirkegaard & Perry Laboratories, Gaithersburg, MD) added; image development took approximately 5–15 min. Stop solution was added and the absorbance was read on a spectrophotometer reading at 450 nm. All reported values represent the average of two wells of the same sample. Specimens were considered positive if the test sample absorbance was above the cut-off value (COV). The COV was determined averaging the cell lysate half of each plate and adding two standard deviations [8].

### Genomic sequencing

Sequencing of the ROCΔCCP and USA+CCP genomes was carried out using standard protocols of the Genome Sequencer FLX pyrosequencing platform (Roche Applied Science, Indianapolis, IN) using the shotgun sequencing protocol.

### Statistical analysis

As data were not normally distributed, nonparametric statistical analyses were used [45]. Using the Wilcoxon rank-sum test, reported data averages were compared within sample day between the following groups, ROC 2003 vs. ROCΔCCP and USA 2003 vs. USA+CCP. All *P*-values lower than 0.05 were considered significantly different.
